# Examining Transdiagnostic Patterns of Motor Differences: Preliminary Findings From the Consortium for Motor Behavior in Neurodivergence (COMBINE)

**DOI:** 10.1007/s10803-025-07042-0

**Published:** 2025-09-27

**Authors:** Nicholas E. Fears, Priscila M. Tamplain, Haylie L. Miller

**Affiliations:** 1 School of Kinesiology, Louisiana State University, 50 Fieldhouse Dr, Baton Rouge, LA 48109, USA; 2 Department of Kinesiology, University of Texas at Arlington, 501 S. Nedderman Dr, Arlington, TX, USA; 3 School of Kinesiology, University of Michigan, 830 N. University Ave, Ann Arbor, MI, USA

**Keywords:** Motor differences, Autism, Developmental coordination disorder, Childhood apraxia of speech, Attention deficit hyperactivity disorder, Neurodevelopmental conditions

## Abstract

While some diagnostic groups are characterized in terms of motor features, others are at risk of overlooked motor challenges due to emphasis on social-cognitive features. These conditions often co-occur, making it difficult to determine the specific contribution of each to the overall pattern of observed motor challenges across neurodivergence. The COMBINE dataset included 262 cases (216 Male, 46 Female) with one or more neurodevelopmental conditions. We used generalized linear models to assess the effect of each of 4 diagnoses (autism, ADHD, DCD, CAS) on Movement Assessment Battery for Children (2nd edition, MABC-2) total score, Manual Dexterity domain score, Aiming & Catching domain score, and Balance domain score; age; sex, and diagnoses. Movement scores were low in all groups, with 77% of cases in the Red Zone (DCD likely). Diagnosis of DCD predicted lower overall scores and Manual Dexterity scores, diagnosis of ADHD predicted higher overall scores and Balance scores, and diagnosis of autism predicted lower Aiming & Catching scores. These results suggest motor challenges are clinically-significant across several neurodevelopmental conditions, and that some conditions have independent effects on domain-specific motor skills. This study is the first step toward determining whether each neurodevelopmental condition has a unique motor “signature”, or if motor differences are ubiquitous across this population.

Serious motor challenges occur at a higher rate in neurodevelopmental conditions than in neurotypicality (Colizzi et al., 2020; Peralta & Cuesta, 2017). Yet, people with neurodevelopmental conditions experience significant disparities in identification and management of motor challenges based on clinical characteristics, racial/ethnic identity, sex, and age (Ewen & Shapiro, 2005; Farran et al., 2020; Miller et al., 2021; Shaw et al., 2021). These disparities place many at high risk for limited functional mobility and physical and behavioral health issues (Hand et al., 2020; Hsieh et al., 2012). It is important to establish an efficient, uniform pipeline for assessment, diagnosis, and intervention to reduce health disparities among people with neurodevelopmental conditions (Burke et al., 2019). The first step in achieving these goals is to identify the behavioral features that overlap or vary between and within diagnostic groups.

Some small, single- or dual-population studies describe overlap and variability in specific motor-related features. For example, motor differences are a key feature of childhood apraxia of speech (CAS), with as many as 85% of people with CAS exhibiting motor challenges significant enough to warrant co-diagnosis of developmental coordination disorder (DCD) (Mandelli et al., 2024). However, clear discrepancies exist between results of objective motor testing like the Movement Assessment Battery for Children – 2nd edition (MABC-2; Henderson et al., 2007) and caregiver reports of motor ability like the Developmental Coordination Disorder Questionnaire (DCD-Q; Wilson et al., 2009). Even though many children with CAS have MABC-2 scores that suggest the presence of significant motor differences, especially in the Manual Dexterity domain, only about half of caregivers endorse functional motor challenges on the DCD-Q (Mandelli et al., 2024). These results emphasize the importance of observational assessment in this population and others where diagnostic overshadowing and caregiver or clinician biases may result in under-estimation of motor difficulties. Fine motor skills are required for school achievement, and problems in this domain may particularly limit access to curricula. The extant literature suggests that different neurodevelopmental conditions may be characterized by problems in specific areas of motor function. It is crucial to examine these patterns transdiagnostically in order to determine whether unique motor “signatures” are present for different groups.

Both neuroimaging and behavioral data suggest motor-related differences between autism, CAS, and neurotypical development. Autistic people’s brains are characterized by greater atypical structure and function in motor-related regions (e.g., basal ganglia, cerebellum) compared to those with CAS, whose brains are characterized by greater atypicality in fronto-temporal regions (Conti et al., 2020). While autistic people experience a wide range of motor behavior differences compared to neurotypical development (Kangarani-Farahani et al., 2024), the available literature on differences between neurotypicality and CAS focuses largely on oral-motor skills (Chilosi et al., 2022; Tükel et al., 2015). Thus, not only do autism and CAS differ in their neurological and behavioral-level motor features compared to neurotypicality, they likely also differ from each other in their underlying etiology and functional output, highlighting the diversity of motor profiles within the neurodivergent community.

A similar but non-identical pattern emerges when comparing DCD, autism, and neurotypicality. Divergent patterns of atypical neurological structure and function are evident when comparing autism to DCD, particularly in areas related to motor imagery (Caeyenberghs et al., 2016; Kilroy et al., 2021). Behaviorally, the degree of motor differences appears similar in autism and DCD when measured only using total scores on common standardized assessments and questionnaires (e.g., MABC-2, DCD-Q; Miller et al., 2021). However, examination of domain-specific scores suggests subtle differences, perhaps implicating underlying differences in neuromotor mechanisms (Miller et al., 2019). Further complicating the landscape, there is notable heterogeneity in autism, posing an interesting challenge for researchers seeking to define boundaries between diagnoses (Waterhouse, 2022).

Some forms of neurodivergence are canonically-motoric in nature (e.g., DCD, CAS) or have motor-specific features (e.g., repetitive motor stereotypies in autism). Others like attention deficit hyperactivity disorder (ADHD) are more often thought of in terms of their cognitive features, even though diagnostic elements like hyperactivity affect both cognitive and motor domains of function. Yet, about half of children with ADHD have clinically-significant motor challenges in some (but not all) domains (Albajara Sáenz et al., 2021; Farran et al., 2020; Reiersen et al., 2008). These motor challenges seem to have a distinct, though not entirely different, neural underpinning compared to other neurodevelopmental conditions (Albajara Sáenz et al., 2021). Co-occurrence of autism and ADHD produces a particularly complex profile, including motor skill variability (Albajara Sáenz et al., 2021; Takagi et al., 2022). Behavioral and neuroimaging work suggests that motor coordination may be a feature of autism that distinguishes it from ADHD alone, where temporal features are more prominent (Albajara Sáenz et al., 2021; Reiersen et al., 2008; Takagi et al., 2022).

Despite serving as a useful starting point for determining the overlap and variability in motor behavior among neurodevelopmental conditions, most prior studies focus on a single diagnostic group or a two-group comparison. This limits our ability to identify feature overlap across conditions. Through transdiagnostic analysis of motor behavior data, we can gain more ecologically-valid insight into how the presence or absence of a particular condition affects motor function, and how motor differences produce functional challenges in individuals with more than one neurodevelopmental condition. Existing transdiagnostic approaches to the biobehavioral taxonomy of neurodivergence conditions, like the Research Domain Criteria (RDoC; Insel et al., 2010) framework, can help to guide our understanding of how motor problems are situated within the larger context of lived experience and physiology (Fritze et al., 2024; Michelini et al., 2024; Morris et al., 2022). In particular, the presence of a specific motor domain in the RDoC framework offers a clear pathway to understanding motoric elements of neurodivergence between and within diagnostic groups, especially as it relates to shared neurophysiology (Hirjak et al., 2018; Kent et al., 2024; Peralta et al., 2017). It also offers a useful means of examining neurodevelopmental traits that can be measured in both motor and non-motor domains, like impulsivity (Zhong et al., 2025), and of characterizing heterogeneity in conditions like autism, where individual profiles of ability are complex and highly variable (Chetcuti et al., 2025).

Most prior studies also focused on early development and typically do not assess multiple developmental timepoints, neglecting the role of lifespan changes in motor behavior. These gaps limit our ability to develop targeted screening, diagnosis, and intervention/accommodation approaches that improve physical health risks(Tyler et al., 2014; Vohra et al., 2016) (e.g., obesity, falls), mental health risks(Pimenta et al., 2023; Tamplain & Miller, 2021) (e.g., depression, low self-esteem) and participation and mobility barriers(Bertilsson et al., 2018; Chatterjee et al., 2023; Gowen et al., 2023; Oliveira et al., 2023; Welch et al., 2021) associated with motor challenges. Data sharing can close important gaps in identification of multi-domain patterns of developmental variability between or within conditions (Astle et al., 2022; Astle & Fletcher-Watson, 2020; Roefs et al., 2022).

The current study uses the Consortium for Motor Behavior in Neurodivergence (COMBINE) (Miller et al., 2022) dataset to extend the prior literature by examining motor skills across people with one or more neurodevelopmental conditions on the same measures. This study uses cross-sectional data obtained from people at various developmental timepoints across childhood and adolescence, and the long-term goal of the COMBINE dataset is to represent both cross-sectional and longitudinal data from participants across the lifespan. COMBINE houses a large, transdiagnostic, lifespan set of multimodal data from sites around the U.S. that can be used to quantify neurodivergent motor behavior and its relation to associated features across the lifespan. In the current study, we examine the relation between different diagnoses (i.e., Autism, ADHD, DCD, CAS) and motor skill outcomes in children and adolescents. We specifically sought to test the independent effects of each diagnosis on specific domains of motor difference observed in neurodivergence, as measured by the MABC-2.

## Method

### Participants

**Fig. 1:**
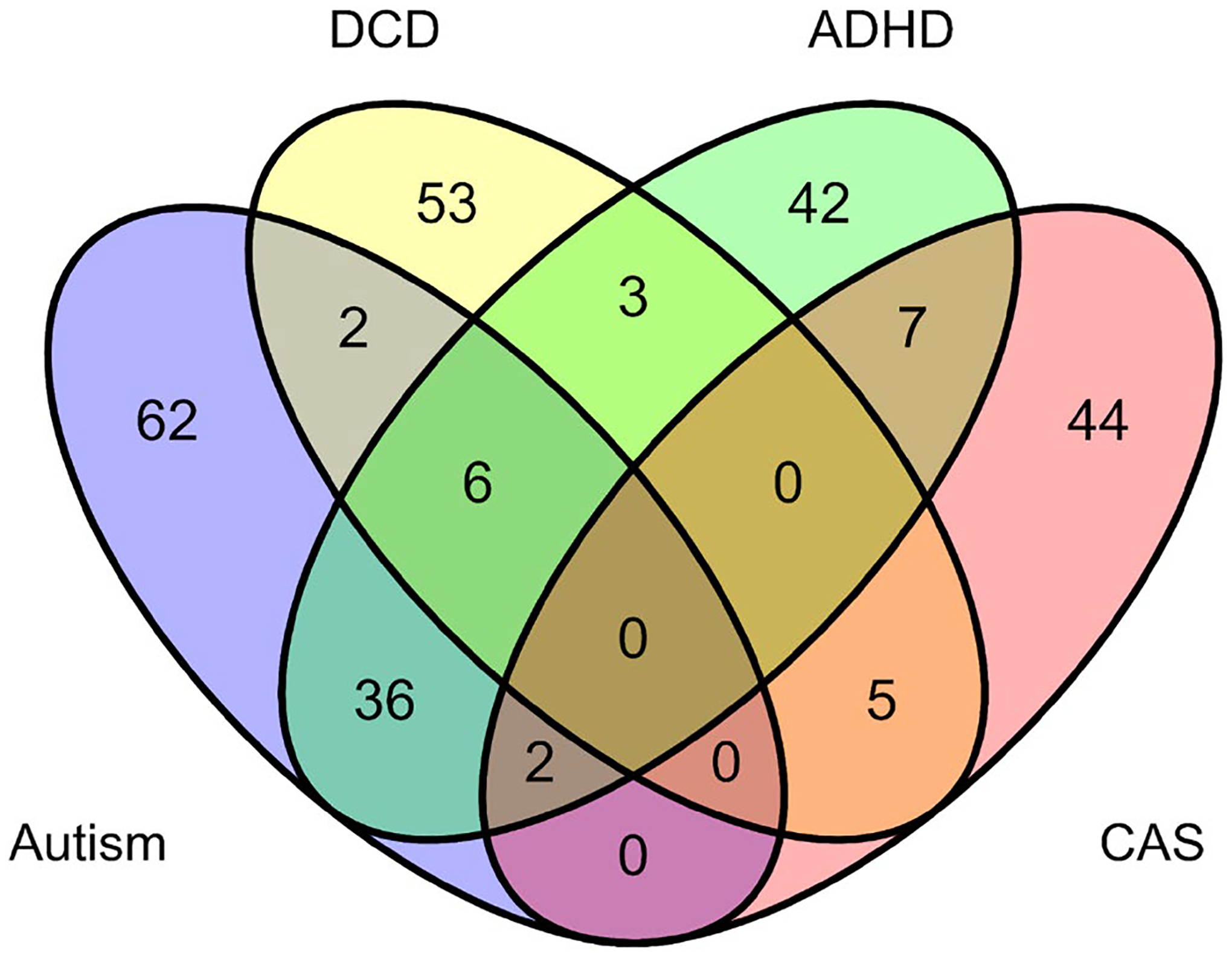
Number of unique cases in the current sample for each diagnostic group: autism (purple), developmental coordination disorder (DCD, yellow), attention deficit hyperactivity disorder (ADHD, green), childhood apraxia of speech (CAS, pink), and multimorbid (overlapping areas)

The current study includes 262 cases (216 Male, 46 Female) representing 4 diagnostic groups of interest: autism, ADHD, DCD, and CAS. There were 56 cases with multiple diagnoses (Fig. 1).

### Procedure

The data for the current study were collected at three independent sites: the University of Texas at Arlington, University of North Texas Health Science Center, and the University of Michigan. Data sharing and use agreements were executed between each contributing institution and the University of Michigan (the coordinating site for COMBINE), and each principal investigator was required to attest that the original study under which the data were collected was approved by their institutional review board, allowed sharing of deidentified study data, and was not subject to additional regulation that may prevent contribution to COMBINE. Prior to depositing data into the COMBINE dataset, principal investigators were required to remove all identifiers.

Principal investigators were provided with a custom data deposit template and codebook to assist with data gathering and harmonization across sites. Data were uploaded to a secure REDCap project used to aggregate and manage COMBINE contributions. REDCap is a secure, web-based software platform designed to support data capture for research studies, providing (1) an intuitive interface for validated data capture; (2) audit trails for tracking data manipulation and export procedures; (3) automated export procedures for seamless data downloads to common statistical packages; and (4) procedures for data integration and interoperability with external sources (Harris et al., 2009, 2019). The REDCap architecture used to host the COMBINE project is maintained by the University of Michigan (the coordinating site for COMBINE).

Data deposited into the COMBINE dataset were validated for acceptable values prior to being imported to REDCap using custom Python code and harmonized in REDCap using 6 variables: MABC-2 total standard score, Manual Dexterity domain standard score, Aiming & Catching domain standard score, and Balance domain standard score; age at date of testing (in months); sex assigned at birth, and diagnoses. All code used to harmonize, validate, and examine data were deposited to a GitHub repository maintained by the research team and available to COMBINE members. Cases with missing age, birth sex, or MABC-2 data were removed from the analyses.

### Measures

*Movement Assessment Battery for Children*, *Second Edition* (MABC-2) (Henderson et al., 2007). This tool is a well-documented, individually administered, standardized test that provides an assessment for children with motor impairment. The MABC-2 is normed for children ranging in age from 3 to 16 years and consists of three age bands: 3–6 years (*n* = 26), 7–10 years (*n* = 70), and 11–16 years (*n* = 23). The test contains 8 subtests across 3 components: Manual Dexterity, Aiming & Catching, and Balance. Each subtest raw score is converted to an item standard score, which accounts for age and test band. Item standard scores are then combined to produce each of the 3 component scores, which are converted into component standard scores. Component scores are added to produce a total test score, which is converted into a total standard score.

Total standard score is classified into three zones: Green (16th percentile or above; total score > 67; DCD unlikely), Amber (6th-15th percentile; total score 57–67; Monitor for DCD) and Red (5th percentile or below; total score < 57; DCD likely). Children and adolescents scoring in the Amber (*n* = 13) or Red (*n* = 106) zones have clinically significant motor impairment. With good reliability (minimum test-retest value at any age is 0.75 and the interrater value is 0.70) and concurrent validity, the MABC-2 is frequently used to identify children with movement difficulties, and it is also commonly used as a tool for eligibility for DCD in research (Blank et al., 2019).

### Data Analysis

We used generalized linear models to capitalize on the transdiagnostic nature of the dataset and enable us to examine the effect of each diagnosis on motor skills as measured by the MABC-2. We regressed MABC-2 Movement Total Standard and Component Standard Scores on age (months), sex assigned at birth, autism diagnosis, ADHD diagnosis, CAS diagnosis, DCD diagnosis as shown in (1). Each of the models is testing the effect of having a specific diagnosis (e.g., ADHD) or not on the dependent variable (e.g., motor skills). For example, the Autism Diagnosis predictor is testing whether neurodivergent children with an autism diagnosis are different from the neurodivergent children without an autism diagnosis. Model assumptions regarding normality and heteroskedasticity were checked via visual examination of *q-q* and residual plots for each model. The continuous variable of age was centered on the mean. We conducted *z-tests* for model coefficients for negative binomial distributions and *t-*tests for model coefficients for gaussian distributions. Means (*M*), standard deviations (*SD*), estimated marginal means (*EMM*) and standard errors (*SE*) are in response scale and *β* weights are reported in the transformed scale.


(1) $$\begin{array}{c} \text{Standard}\;\text{Score}=\beta_{0}+\beta_{1}Age+\beta_{2}\text{BirthSex}+\beta_{3}\text{Autism}+\beta_{4}\text{ADHD}+\beta_{6}CAS+\beta_{6}DCD \end{array}$$

## Results

**Fig. 2:**
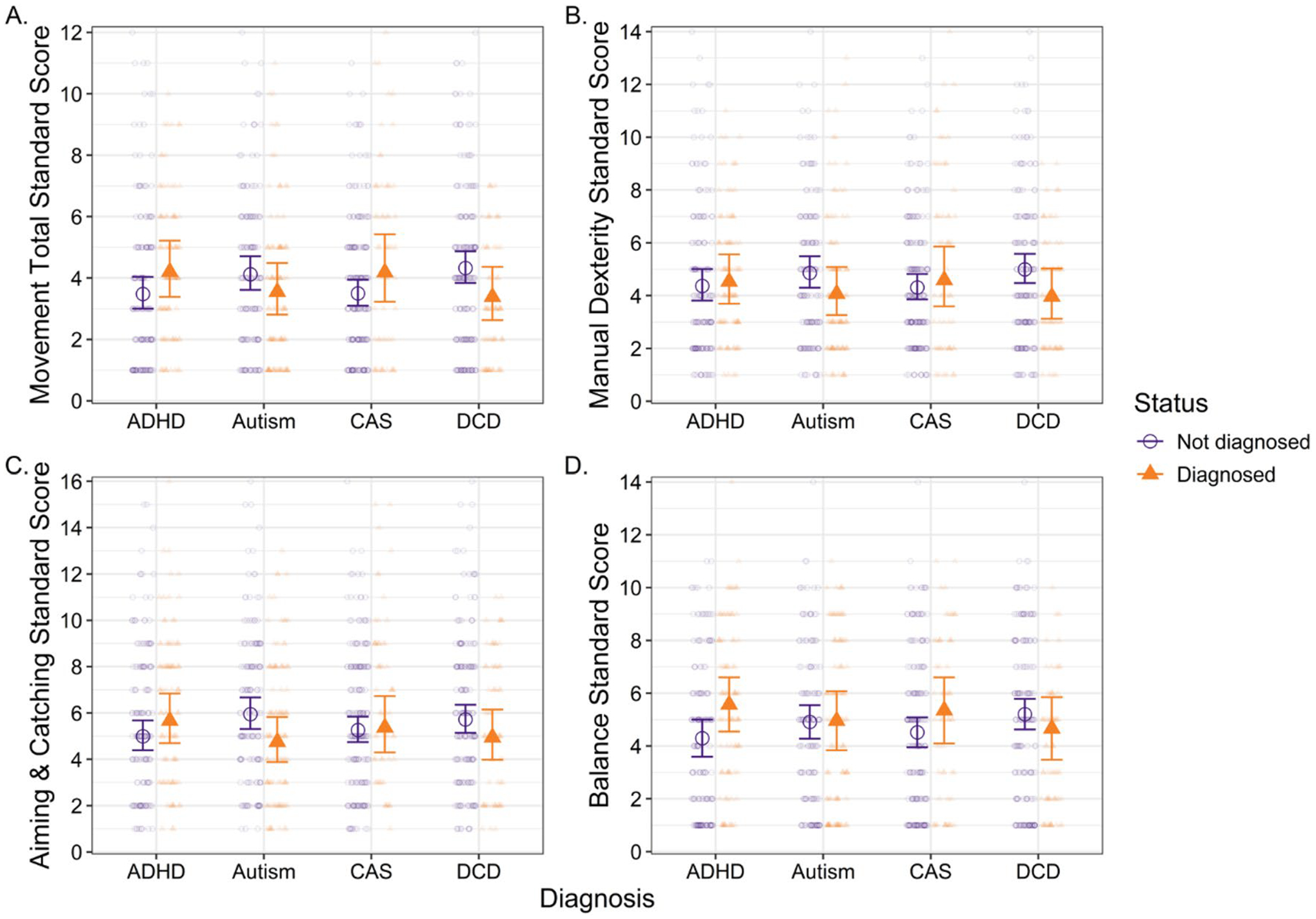
Standard scores for the **a** Movement Total, **b** Manual Dexterity Composite, **c** Aiming & Catching Composite, and **d** Balance Composite of the Movement Assessment Battery for Children, 2nd Edi tion. *ADHD* Attention Deficit-Hyperactivity Disorder; *CAS* Childhood Apraxia of Speech; *DCD* Developmental Coordination Disorder

Cases in the final sample had an age range of 37 to 198.6 months (M = 108.9, SD = 39.0, median = 101.1). Across the sample, MABC-2 Movement Total Standard scores were low (M = 3.89, SD = 2.49, median = 4, range = 1–12), with 201 cases (77%) in the Red Zone (i.e., DCD likely), 22 cases (8%) in the Amber Zone (i.e., monitor for DCD), and 39 cases (15%) in the Green Zone (i.e., DCD unlikely). For MABC-2 Standard Scores by diagnostic group, see Fig. 2 and Table 1.

### Movement Total Standard Scores

We regressed MABC-2 Movement Total Standard scores on age, birth sex, and each of the listed diagnoses using a generalized linear model using a negative binomial distribution (Table 2). We found significant effects of DCD diagnosis (*β* =−0.245, *p* = .034) and ADHD diagnosis (*β* =0.189, *p*=.039), indicating a diagnosis of DCD (*EMM*=3.38, *SE* = 0.44) predicted lower scores compared to not having a DCD diagnosis (*EMM* = 4.32, *SE* = 0.26). A diagnosis of ADHD (*EMM*=4.20, *SE* = 0.46) predicted higher Movement Total Standard Scores compared to not having an ADHD (*EMM*=3.48, *SE* = 0.26).

### Manual Dexterity Composite Standard Scores

We regressed MABC-2 Manual Dexterity Composite Standard scores on age, birth sex, and each of the listed diagnoses using a generalized linear model using a negative binomial distribution (Table 3). We found a significant effect of DCD diagnosis (*β* =−0.232, *p* = .031), indicating a diagnosis of DCD (*EMM* = 3.96, *SE* = 0.48) predicted lower scores compared to not having a DCD diagnosis (*EMM* = 4.99, *SE* = 0.28).

### Aiming & Catching Composite Standard Scores

We regressed MABC-2 Aiming & Catching Composite Standard scores on age, birth sex, and each of the listed diagnoses using a generalized linear model using a negative binomial distribution (Table 4). We found a significant effect of autism diagnosis (*β* =−0.225, *p* = .010) indicating an autism diagnosis (*EMM* = 4.75, *SE* = 0.49) predicted lower scores compared to not having an autism diagnosis (*EMM* = 5.94, *SE* = 0.35). We also found a significant effect of birth sex (*β* =−0.199, *p* = .025), indicating that being assigned female (*EMM* = 4.81, *SE* = 0.48) at birth compared to male predicted lower scores (*EMM* = 5.87, *SE* = 0.39).

### Balance Composite Standard Scores

We regressed MABC-2 Balance Composite Standard scores on age, birth sex, and each of the listed diagnoses using a generalized linear model using a Gaussian distribution with an identity link (Table 5). We found significant effects of ADHD diagnosis (*β* =1.28, *p*=.003), indicating a diagnosis of ADHD (*EMM*=5.57, *SE* = 0.52) predicted higher scores compared to not having an ADHD diagnosis (*EMM*=4.29, *SE* = 0.36).

## Discussion

In this initial analysis of the COMBINE dataset, we examined the relationship between different diagnoses (i.e., autism, ADHD, DCD, CAS) and motor skill outcomes in children and adolescents. We specifically sought to test the independent effects of each diagnosis on MABC-2 total and domain-specific scores. Generally, having a diagnosis of DCD predicted lower total scores and having a diagnosis of ADHD predicted higher total scores compared to those without. No age differences were found, but being assigned female at birth was associated with lower Aiming & Catching domain scores.

### Domain-specific Performance Differences by Diagnosis

Diagnostic group also predicted performance on domain scores. Specifically, autistic children had significantly lower Aiming & Catching domain scores compared to those without an autism diagnosis. This finding aligns with previous literature indicating that motor challenges in autism may stem from visuomotor integration differences (Lepping et al., 2022; Miller et al., 2014, 2023). These domain-specific difficulties may differentiate autistic individuals from those without an autism diagnosis (Caçola et al., 2017; Miller et al., 2019). Future research should further investigate the underlying mechanisms of visuomotor integration difficulties in autism and its utility in differential diagnosis of motor difficulties in children with neurodevelopmental conditions.

Children with a DCD diagnosis had significantly lower Manual Dexterity scores compared to children without a DCD diagnosis. Problems with Manual Dexterity can negatively affect children’s academic success and self-esteem (Feder & Majnemer, 2007), potentially prompting families to seek additional motor assessment through the school system that results in a DCD diagnosis. Notably, although the presence of a DCD diagnosis uniquely predicted lower Manual Dexterity scores, scores in this domain were low across the entire sample. This underscores the importance of universal motor assessment for children with neurodevelopmental conditions. Specifically, assessments for educational accommodations and interventions should, at the very least, assess Manual Dexterity as part of a systematic evaluation of needs.

### Diagnostic Overlap and Risk of Overshadowing

Clinically, motor challenges are either described as primary (DCD) or associated (autism, CAS, and ADHD) features of neurodevelopmental conditions (American Psychological Association., 2012; World Health Organization., 2019). For conditions less-commonly characterized in terms of their motor features (e.g., autism, ADHD), there remains ongoing discussion about whether motor challenges are a core diagnostic feature with condition-specific presentation, or reflective of co-occurring DCD (Miller et al., 2024). The first results of the COMBINE dataset support the notion that motor challenges are prevalent across several neurodevelopmental diagnoses, and that they are significant enough to warrant assessment and management.

For example, although having a diagnosis of ADHD predicted higher total scores and Balance domain scores than not having an ADHD diagnosis in this sample, more than 80% of children with an ADHD diagnosis in this sample had or were at risk of significant motor challenges. While motor challenges in ADHD might not be as pronounced relative to those of people with other neurodevelopmental conditions, they manifest to a degree that is likely to significantly affect functional movement. This again underscores the importance of assessing motor skills across all neurodevelopmental conditions, even those not commonly characterized in terms of their motor features.

In addition, of the 39 cases in the COMBINE dataset who scored in the Green Zone, 17 were in the 16th percentile, which may also be considered the upper end of the Amber Zone, denoting a risk for movement difficulty and monitoring required (according to the MABC-2 manual). We were conservative with our results, but it is alarming that 17 cases could be classified as having no motor difficulty despite scoring barely within the threshold. A child with high day-to-day variability in support needs, as is common in neurodevelopmental conditions (Hadwin et al., 2023; Mandelli et al., 2024; Parma & de Marchena, 2016; Smits-Engelsman & Wilson, 2013), might then be denied the opportunity for referral, evaluation, and intervention. Assigning a diagnosis of DCD when existing can support several pathways for intervention and accommodations in the school environment, and can increase parental understanding of how to effectively support their children (Klein et al., 2024; Materula et al., 2024).

Further work is needed to fully characterize transdiagnostic patterns of motor difference in neurodivergence and determine their relation to core diagnostic features. Comparison to typical development is not always necessary to answer these open questions–rather, comparison among neurodevelopmental conditions may provide more clinically-relevant context for understanding how motor differences and challenges relate to best practices for diagnosis and management.

Importantly, the present study also elucidates the fact that diagnostic lines between neurodevelopmental conditions are not black and white, as many individuals have more than one co-occurring condition in addition to their so-called “primary” diagnosis (e.g., autism with co-occurring ADHD). Our analytic approach enabled us to focus on the additive effect of each diagnosis individually, while maintaining a transdiagnostic lens to maximize ecological validity. These results begin to shed light on how the presence or absence of a given diagnosis may relate to Manual Dexterity, Aiming & Catching, and Balance. By parsing the independent effects of each diagnosis, we can begin to determine whether each group has its own unique motor “signature”, or if motor differences are uniform across neurodevelopmental conditions.

### Limitations and Future Directions

Although data were collected across multiple groups, which could affect reliability, these standardized clinical assessments were designed for use across diverse research, clinical, and educational settings (Brown & Lalor, 2009; Cools et al., 2009). The preliminary set of data included in this study were collected from sites where the researchers had extensive experience administering motor assessments to neurodivergent individuals.

As COMBINE grows, there will likely be greater variability in the degree of training and expertise of contributing investigators and their teams. For that reason, the COMBINE intake process includes collection of investigator credentials and a discussion about experience and standardization to better contextualize future analyses where variability in administrator expertise could affect outcomes. While there is not a formal training requirement for administration of the MABC, the most recent edition (MABC-3) offers a complimentary Pearson webinar and a resource library of videos to support training and standardized administration. In our view, the goal of increasing data sharing and assessment and intervention for motor difficulties in neurodivergent individuals outweighs the potential risk of minor measurement error across sites.

We suggest that the MABC has more strengths than weaknesses with respect to the goal of identifying motor problems among neurodivergent people. These strengths include include a straightforward administration procedure, detailed instructions provided in the manual, tasks that are accessible to participants at many levels of cognitive and physical ability, and scoring criteria that are objective and clear for administrators at many levels of expertise.

COMBINE is designed to prioritize motor assessment and to reduce barriers to data sharing, and so contributors are not required to administer a uniform battery of developmental assessments. This structure is somewhat different from other data repositories and comes with both advantages and risks. We rely on sites to have the appropriate expertise and procedures in place to establish or confirm diagnoses for the populations they work with, as this is a professional expectation of both researchers and clinicians. COMBINE contributors are asked to only affirm those diagnoses for which DSM or ICD criteria were met based on (1) assessment or confirmation performed within the research team, or (2) prior assessment by a clinical or educational professional outside of the research team. However, known issues of diagnostic overshadowing may lead to missing information about possible co-occurrence that could have been captured with a comprehensive developmental assessment.

While the COMBINE dataset is substantially larger and more diverse than typical studies of neurodevelopmental conditions, the cell size for each combination of co-occurring diagnoses remains relatively small and many conditions are not yet represented. For example, the current sample of children with CAS had a lower incidence of co-occurring diagnoses (e.g., autism) than needed to meaningfully characterize transdiagnostic patterns, particularly given prevalence estimates. Additionally, some neurodevelopmental conditions were not yet represented in the dataset at the time of this preliminary analysis (e.g., cerebral palsy, intellectual disability, dyslexia).

Relying on binary diagnostic variables (i.e., presence/absence of autism based on DSM or ICD criteria) also provides a less-nuanced means of examining neurodivergence than the use of continuous measures of different neurodevelopmental traits. As COMBINE continues to grow, contributors are encouraged to deposit as much continuous data as possible from measures of other neurodevelopmental domains (e.g., attention, language, social communication skills), so that future analyses can better represent the dimensionality of neurodevelopmental traits observed within the neurodivergent population, in alignment with the RDoC framework. Our long-term goal is to compile the larger, more diverse sample needed to analyze data in a more nuanced, dimensional manner across neurodevelopmental conditions, facilitating a truly transdiagnostic approach to understanding neurodivergent motor behavior.

In alignment with the goal of expanding representation within the COMBINE dataset, recently the third edition of the MABC (MABC-3; Henderson & Barnett, 2007) extended the upper age limit from 16 years to 25 years of age. This change allows for continuity of motor skill assessment from 3 years of age through young adulthood. This is an important advancement, given the paucity of research on motor difficulties in young and middle adulthood among people with neurodevelopmental conditions. Even though the MABC-3 likely has some of the same limitations identified in the previous edition, such as ceiling effects (French et al., 2018) and misrepresentations due to several factors such as variations in mental and physical state and lack of formal examiner training (Hadwin et al., 2023), it continues to offer a direct and reasonable means of assessing motor performance across a wide variety of professional settings globally (Hadwin et al., 2023). A single lab may not have the time and funds to collect samples of the size and diversity necessary to investigate patterns across the entire landscape of neurodivergent motor development, but these questions can be answered through data-sharing efforts when measures are selected with harmonization in mind. Large, global data-sharing consortia like COMBINE offer an opportunity to characterize transdiagnostic patterns of motor behavior in neurodivergence that may be meaningful in development of policy and practice guidelines.

## Conclusions

We conclude that significant motor challenges are present across each of the conditions we examined in the present study (ADHD, autism, CAS, DCD). Our analytic approach enabled us to identify several unique effects of having or not having a given diagnosis on motor skills. This yielded important information about the role of ADHD and DCD in predicting higher and lower total movement scores, respectively, as well as some diagnosis-specific effects on the domains of Manual Dexterity, Aiming & Catching, and Balance. These first results from the COMBINE dataset highlight the utility of transdiagnostic analysis, and support continued expansion of the consortium to include a broader range of conditions and ages.

## Figures and Tables

**Table 1:** Age (mos) and MABC-2 Movement Total Standard Scores and Zones by Diagnostic Group

Diagnostic Group	*n*	M_age_ (SD_age_)	Range_age_	M_mabc_ (SD_mabc_)	Range_mabc_	Red Zone *n* (%)	Amber Zone *n* (%)	Green Zone *n* (%)
Autism	108	124.4 (38.3)	60.0–198.6	3.6 (2.4)	1–11	91 (84.26%)	3 (2.78%)	14 (12.96%)*
ADHD	96	122.0 (35.1)	60.0–194.3	4.4 (2.2)	1–10	68 (70.83%)	12 (12.50%)	16 (16.67%)*
DCD	69	109.0 (37.6)	47.0–190.0	3.1 (1.9)	1–7	58 (84.06%)	7 (10.14%)	4 (5.80%)*
CAS	58	73.5 (22.3)	37.0–154.0	4.7 (3.1)	1–12	34 (58.62%)	7 (12.07%)	17 (29.31%)*

Note. Diagnostic groups are not mutually-exclusive; participants may be represented in multiple rows due to co-occurring diagnoses

* 6 members of the autism group, 4 members of the DCD group, 7 members of the ADHD group, and 8 members of the CAS group scored at the 16th percentile. There is some ambiguity in MABC-2 scoring procedures regarding whether this percentile falls within the Amber or Green Zone. For the purpose of this study, we have classified these cases into the Green Zone

**Table 2:** Summary of GLM for movement total standard scores

	Estimate	Standard Error	z value	*p*-value	
(Intercept)	1.36	0.11	12.43	0.01	***
Age (mos)	− 0.00	0.00	− 0.16	0.88	
Birth Sex	− 0.01	0.10	− 0.10	0.92	
Autism Diagnosis	− 0.15	0.10	− 1.47	0.14	
DCD Diagnosis	− 0.25	0.12	− 2.12	0.03	*
ADHD Diagnosis	0.19	0.09	2.06	0.04	*
CAS Diagnosis	0.18	0.12	1.46	0.14	

Signif. codes: 0 <= *** < 0.001 < ** < 0.01 < * < 0.05

**Table 3:** Summary of GLM for manual dexterity standard scores

	Estimate	Standard Error	z value	*p* value	
(Intercept)	1.61	0.10	15.69	0.00	***
Age (mos)	− 0.00	0.00	− 0.37	0.71	
Birth Sex	0.08	0.09	0.82	0.41	
Autism Diagnosis	− 0.18	0.10	− 1.84	0.07	
DCD Diagnosis	− 0.23	0.11	−2.16	0.03	*
ADHD Diagnosis	0.04	0.09	0.43	0.67	
CAS Diagnosis	0.06	0.12	0.54	0.59	

Signif. codes: 0 <= *** < 0.001 < ** < 0.01 < * < 0.05

**Table 4:** Summary of GLM for aiming & catching standard scores

	Estimate	Standard Error	z value	*p* value	
(Intercept)	1.88	0.09	20.15	0.00	***
Age (mos)	− 0.00	0.00	− 0.63	0.53	
Birth Sex	− 0.20	0.09	− 2.24	0.03	*
Autism Diagnosis	− 0.22	0.09	− 2.57	0.01	*
DCD Diagnosis	− 0.14	0.10	− 1.48	0.14	
ADHD Diagnosis	0.13	0.08	1.62	0.10	
CAS Diagnosis	0.02	0.11	0.20	0.84	

Signif. codes: 0 <= *** < 0.001 < ** < 0.01 < * < 0.05

**Table 5:** Summary of GLM for balance standard scores

	Estimate	Standard Error	z value	*p* value	
(Intercept)	4.19	0.52	8.07	0.00	***
Age (mos)	− 0.00	0.01	− 0.08	0.94	
Birth Sex	− 0.12	0.48	− 0.26	0.80	
Autism Diagnosis	0.04	0.49	0.09	0.93	
DCD Diagnosis	− 0.54	0.54	− 1.01	0.31	
ADHD Diagnosis	1.28	0.43	2.95	0.00	**
CAS Diagnosis	0.84	0.60	1.39	0.16	

Signif. codes: 0 <= *** < 0.001 < ** < 0.01 < * < 0.05
